# Excision of a Solitary Neurofibroma in the Right Masseter Muscle via a High Perimandibular Approach: A Case Report and Review of the Literature

**DOI:** 10.1155/crid/8838385

**Published:** 2025-07-05

**Authors:** Anna Tomimatsu, Atsushi Kasamatsu, Masumi Igari, Chisato Hashimoto, Shuko Koide, Yutaro Kase, Manabu Iyoda, Dai Nakashima, Katsuhiro Uzawa

**Affiliations:** ^1^Department of Oral Science, Graduate School of Medicine, Chiba University, Chiba, Japan; ^2^Department of Dentistry and Oral-Maxillofacial Surgery, Chiba University Hospital, Chiba, Japan

**Keywords:** case report, high perimandibular approach, masseter muscle, neurofibroma

## Abstract

We present a case of solitary neurofibroma in the masseter muscle that was excised via the high perimandibular approach (HPMA). A 52-year-old Japanese man complained of an uncomfortable feeling in his right buccal region. On examination, the buccal mucosa appeared normal, and overall, there were no skin disorders on the patient's body. Computed tomography revealed a well-defined 33 × 22-mm mass in the right masseter muscle showing a low density in the center and a slightly higher density at the margin of the mass. Magnetic resonance imaging also showed a hyperintense area on T1-weighted and T2-weighted images in the masseter muscle. An excisional biopsy was scheduled for the diagnosis of the intramasseteric mass. It was performed via the HPMA with the patient under general anesthesia. The final diagnosis was a neurofibroma. The patient recovered after the surgery without postoperative complications, including facial nerve damage and trismus. He was satisfied with the esthetic outcome, and the tumor has not recurred over a 24-month follow-up period. Altogether, these outcomes indicate that the HPMA is an effective and useful option for excision of a tumor in the masseter muscle.

## 1. Introduction

A neurofibroma is a benign tumor of the peripheral nerve that is composed of a variable mixture of Schwann cells, perineurial-like cells, and fibroblasts. It is known to be a manifestation of the von Recklinghausen disease (neurofibromatosis Type 1; NF1) [1]. The frequency of solitary neurofibromas occurring in the oral cavity is reported to be approximately 6.5% [2]. A common site of neurofibroma in the oral cavity is the tongue [3], whereas its occurrence in the masseter muscle is quite rare.

Intramasseteric tumors have been excised via several approaches, including the submandibular approach, the pre/intra-auricular approach, the retromandibular approach, the high perimandibular approach (HPMA), and the intraoral approach [4, 5]. Each approach presents both advantages and disadvantages for approaching the intramasseteric mass, securing the operative field, and risking injury to the facial nerve [6]. Among the approaches, there have been a few published reports on the use of HPMA for the excision of intramasseteric tumors [7–9]. Here, we present a case of solitary neurofibroma in the masseter muscle that was excised via the HPMA and provide a review of the clinical approaches to intramasseteric tumors.

## 2. Case Report

A 52-year-old Japanese man was referred to our clinic with the complaint of an uncomfortable feeling in the right buccal region. The medical examination found a painless, slightly swollen area in the right buccal region. There were no palpable lymph nodes bilaterally in the submandibular and neck regions. The patient underwent bilateral orthognathic surgery 30 years prior to this clinic visit and did not have a history of systemic diseases.

**Figure 1:**
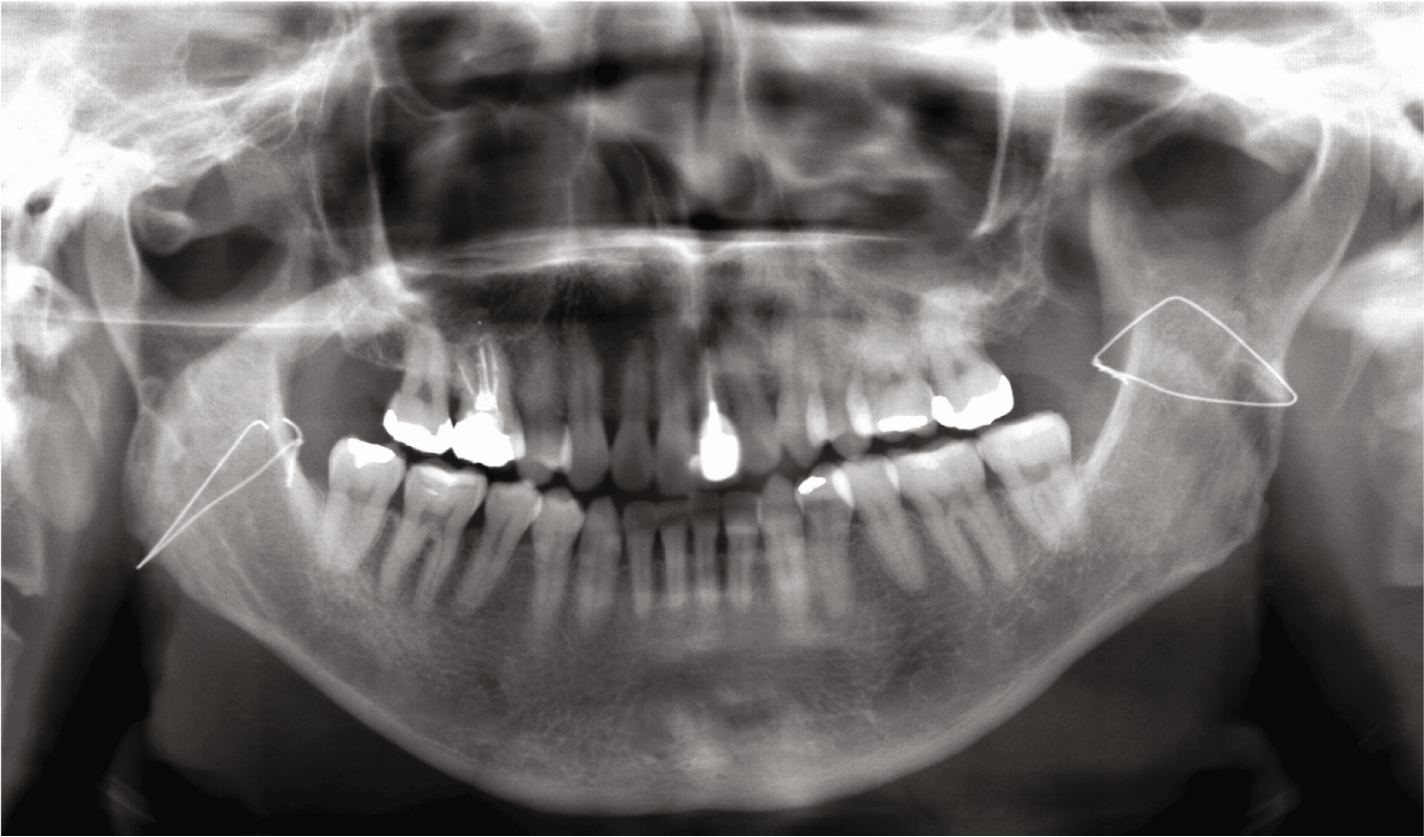
Panoramic radiograph shows normal findings except for the wires of prior orthognathic surgery at the mandibular rami.

**Figure 2:**
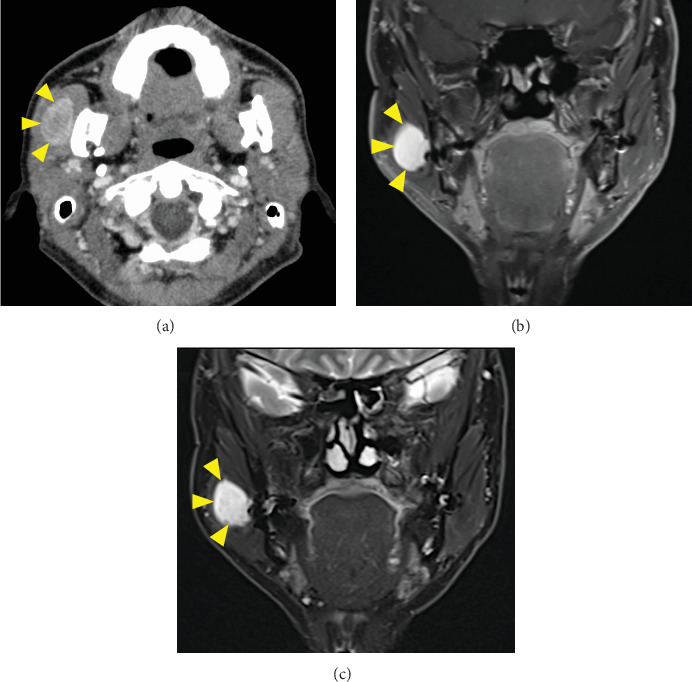
CT and MR images. (a) CT shows a well-defined 33 × 22-mm mass in the right masseter muscle showing low density in the center and slightly higher density at the margins of the mass. (b) T1-weighted and (c) T2-weighted MR images show a high-intensity mass in the right masseter muscle.

Panoramic radiography was negative for abnormal findings. Wires placed during his orthognathic surgery were visible on the mandibular rami (Figure 1). Computed tomography (CT) revealed a well-defined 33 × 22-mm mass in the right masseter muscle showing a low density in the center and a slightly higher density at the margins of the mass (Figure 2a). Magnetic resonance imaging (MRI) also showed a hyperintense mass on T1-weighted (Figure 2b) and T2-weighted images (Figure 2c) in the right masseter muscle. The tumor was isolated, and there were no clinical findings suggesting neurofibromatosis. An excisional biopsy was scheduled for the diagnosis of the intramasseteric mass. The surgery was performed via the HPMA with the patient under general anesthesia.

**Figure 3:**
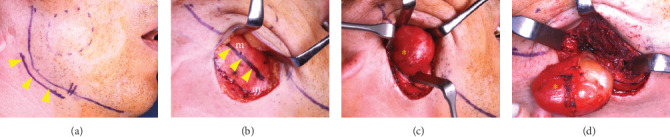
Surgical steps involved in the HPMA. (a) The skin incision for the HPMA is placed 5 mm below and parallel to the edge of the mandibular angle (arrowheads). (b) Skin and subcutaneous tissue are dissected and undermined 2–3 cm upward over the masseter muscle (m). The incision line of the masseter muscle (arrowheads). (c, d) The tumor (asterisk) is exposed and excised.

**Figure 4:**
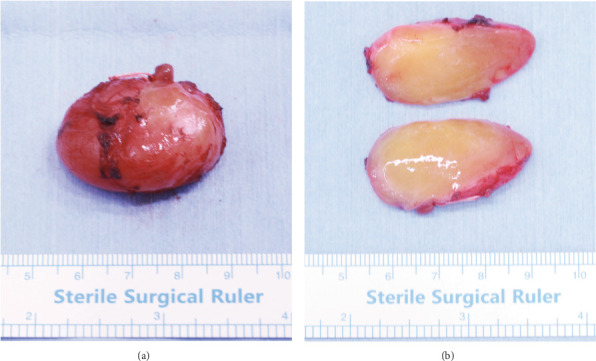
Excised specimen. (a) Gross view of the excised specimen showing a well-circumscribed oval mass measuring 33 × 22 mm. (b) Longitudinal cross-sections of the excised tumor show homogenous, shiny, and light yellow surfaces.

The HPMA was performed according to the Nakaoka method [6]. Briefly, a 5-cm skin incision was placed 5 mm below and parallel to the edge of the mandibular angle (Figure 3a). Skin and subcutaneous tissue were dissected and undermined 2–3 cm upward over the platysma muscle. The platysma muscle was incised to expose the masseter muscle (Figure 3b), and the intramasseteric tumor was then exposed (Figure 3c,d). Finally, the tumor was completely excised from the masseter muscle. Grossly, the excised specimen was a well-circumscribed oval mass measuring 33 × 22 mm. The surfaces of longitudinal cross-sections of the excised tumor appeared homogeneous, shiny, and light yellow (Figure 4a,b).

**Figure 5:**
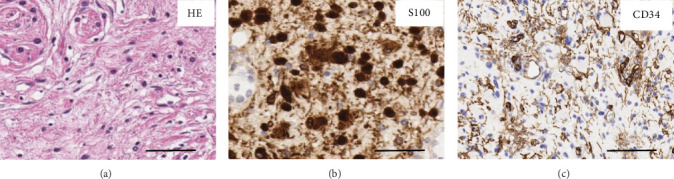
Histopathological findings. (a) HE staining shows small spindle cells, fine fibers, Schwann cells, and occasional mast cells (× 400; scale bar, 50 *μ*m). (b) Immunohistochemical staining for S100 is positive, showing its presence in the cytoplasm and nuclei of the Schwann cells (× 400; scale bar, 50 *μ*m). (c) Immunohistochemical staining for CD34 shows that most tumor cells were diffusely positive (× 400; scale bar, 50 *μ*m).

On histopathological examination, hematoxylin–eosin (HE) staining showed small spindle cells, fine fibers, Schwann cells, and occasional mast cells (Figure 5a, × 400). Immunohistochemical stainings were positive for S100 (Figure 5b) and CD34 (Figure 5c), indicating that the final diagnosis was neurofibroma. The patient's postoperative course was uneventful without complications, including facial nerve damage and trismus. The patient was satisfied with the esthetic outcome, and recurrence has not occurred over a 24-month period after surgery.

## 3. Discussion

NF1 is one of the most common autosomal dominant conditions of the nervous system. Its clinical presentation is highly variable and may include multiple neoplasms, as well as cutaneous, vascular, bony, and cognitive manifestations [10]. The clinical manifestations of NF1 include neurofibromas, café-au-lait spots, freckles on the skin, skeletal dysplasia, Lisch nodules, and optic gliomas. Solitary neurofibromas not associated with NF1 are unlikely to recur and show malignant change [11–13], suggesting that complete excision is important for initial treatment.

Oral neurofibromas are most frequently found in the tongue [3] and rarely on the gingiva, mandible, and floor of the mouth. To date, there is a lack of information on neurofibromas in the masseter muscle, and intramuscular hemangiomas make up the majority of intramasseteric tumors [14].

There are two main objectives involved in the surgical procedures used to excise intramasseteric tumors. First, the surgeons must avoid damage to the facial nerve because the trunk and branches of the facial nerve involve the parotid gland and regions adjacent to the masseter muscle. Second, esthetically, it is important to avoid creating conspicuous postoperative scarring.

Intraoral and extraoral surgical approaches have been reported for tumors in the masseter muscle [15–18]. The intraoral approach avoids visible scarring; however, the operative field is limited, and the surgical procedures are complicated. Extraoral approaches more easily secure the surgical field than the intraoral approaches but are likely to injure the branches of the facial nerve and leave an obvious scar on the face [5].

Hamada and Nakaoka reported that the incidence of facial neuropathy after the HPMA is performed for mandibular fractures is 0%–0.9%, which is extremely low compared with other approaches, such as the retromandibular approach and the Risdon approach [5]. The findings suggest that the HPMA is a suitable technique not only for mandibular fractures but also for intramasseteric tumors. The HPMA through the skin provides the easiest access to intramasseteric tumors, with a wide field of view that allows identification of the mandibular and buccal branches of the facial nerve while the skin flap is elevated [8, 19]. An issue of concern with HPMA is that the scar of the skin incision is located below the edge of the mandibular angle. The postoperative scar has been reported to be inconspicuous by several studies because the scar is hidden by the shadow of the mandibular angle [4, 6, 9].  Table 1 lists the five cases of intramuscular tumors located in the masseter muscle that were excised via the HPMA. There were four cases of hemangioma and our case of neurofibroma [7–9]. None of the cases developed postoperative complications, including facial nerve damage, trismus, and motor dysfunction. Therefore, HPMA is an effective and useful alternative approach for intramasseteric tumors.

## 4. Conclusions

HPMA is a surgical approach for subcondylar fracture of the mandible. Since this approach has been reported to be effective in avoiding damage to the facial nerve and solves esthetic issues, it is also suitable for intramasseteric tumors. The excellent outcomes obtained by the HPMA in our case show that the HPMA may be a useful and convenient approach for excision of intramasseteric neurofibromas.

## Figures and Tables

**Table 1:** Excision of intramasseteric tumors via the HPMA.

**Authors**	**Year**	**Age**	**Sex**	**Tumor size (mm)**	**Main complaint**	**Postoperative complications**	**Final diagnosis**
Jolly et al. [7]	2015	15	F	30 × 20 × 20 CT findings	Swelling in the mandibular angle	—	Hemangioma
Sukedai et al. [8]	2020	46	F	20 CT findings	Slight swelling in the right buccal region	—	Hemangioma
Ishizuka et al. [9]	2022	56	F	11 × 11 × 10 MRI findings	Slight swelling in the left buccal region	—	Hemangioma
Ishizuka et al. [9]	2022	70	F	12 × 10 × 10 MRI findings	Slight swelling in the left buccal region	—	Hemangioma
Present case	2024	52	M	33 × 22 CT findings	Uncomfortable feeling in the right buccal region	—	Neurofibroma

## Data Availability

All data related to the presented manuscript are included within the article.

## References

[1] Komatsu Y., Takeda Y., Kawai T. (2022). A Case of Solitary Neurofibroma in the Maxillary Gingiva. *Journal of Surgical Case Reports*.

[2] Broly E., Lefevre B., Zachar D., Hafian H. (2019). Solitary Neurofibroma of the Floor of the Mouth: Rare Localization at Lingual Nerve With Intraoral Excision. *BMC Oral Health*.

[3] Singh Sahota J., Viswanathan A., Nayak R., Hazarika P. (1996). Giant Neurofibroma of the Tongue. *International Journal of Pediatric Otorhinolaryngology*.

[4] Lutz J. C., Clavert P., Wolfram-Gabel R., Wilk A., Kahn J. L. (2010). Is the High Submandibular Transmasseteric Approach to the Mandibular Condyle Safe for the Inferior Buccal Branch?. *Surgical and Radiologic Anatomy*.

[5] Hamada Y., Nakaoka K. (2020). Current Strategy of Diagnosis and Treatment for Mandibular Condylar fractures. *Japanese Journal of Oral & Maxillofacial Surgery*.

[6] Nakaoka K., Yamada H., Horiuchi T., Nakajima T., Nakatani H., Hamada Y. (2016). Usefulness of a High Perimandibular Approach for Open Reduction and Internal Fixation of Mandibular Condyle Fractures. *Journal of Oral and Maxillofacial Surgery*.

[7] Jolly S. S., Rattan V., Rai S., Kaur K., Gupta A. (2015). Intramuscular Cavernous Haemangioma of Masseter Muscle – A Case Report of Surgical Excision. *Journal of Clinical and Diagnostic Research*.

[8] Sukedai M., Manabe R., Kinoshita Y. (2021). The High Perimandibular Approach for Excision of an Intramasseter Tumor: A Clinical Report. *Journal of Oral and Maxillofacial Surgery, Medicine, and Pathology*.

[9] Ishizuka T., Nakaoka K., Arai G., Eguchi T., Hojo H., Hamada Y. (2022). Two Cases of Surgical Removal of Intramasseter Hemangiomas Using the High Perimandibular Approach. *Japanese Journal of Oral and Maxillofacial Surgery*.

[10] Ly K. I., Blakeley J. O. (2019). The Diagnosis and Management of Neurofibromatosis Type 1. *Medical Clinics of North America*.

[11] Zachariades N., Mizens M., Vairaktaris E. (1987). Benign Neurogenic Tumors of the Oral Cavity. *International Journal of Oral and Maxillofacial Surgery*.

[12] Nagai H., Enakata A., Ehatakeyama S., Fukuda M., Enanjo H., Emiyamoto Y. (2006). A Case of Solitary Neurofibroma in the Cheek. *Japanese Journal of Oral & Maxillofacial Surgery*.

[13] Weaver B. D., Graves R. W., Keyes G. G., Lattanzi D. A. (1991). Central Neurofibroma of the Mandible: Report of a Case. *Journal of Oral and Maxillofacial Surgery*.

[14] Ichimura K., Yanaka T., Kitahara N. (1989). Surgery of the Masseter Muscle. *Practica Oto-Rhino-Laryngologica*.

[15] Tsumuraya G., Yamada H., Shimizu H., Hamada Y. (2014). Intramuscular Lipoma in the Masseter Muscle: A Case Report. *British Journal of Oral and Maxillofacial Surgery*.

[16] Smith W. P., Prince S., Phelan S. (2005). The Role of Imaging and Surgery in the Management of Vascular Tumors of the Masseter Muscle. *Journal of Oral and Maxillofacial Surgery*.

[17] Kanaya H., Saito Y., Gama N., Konno W., Hirabayashi H., Haruna S. (2008). Intramuscular Hemangioma of Masseter Muscle With Prominent Formation of Phleboliths: A Case Report. *Auris Nasus Larynx*.

[18] Surej K. L. K., Kurien N. M., Venugopal K., Nair P. R., Mony V. (2016). Intramuscular Hemangioma of the Masseter Muscle- A Case Report and Review of Literature. *International Journal of Surgery Case Reports*.

[19] Meyer C., Kahn J. L., Boutemi P., Wilk A. (2002). Photoelastic Analysis of Bone Deformation in the Region of the Mandibular Condyle During Mastication. *Journal of Cranio-Maxillofacial Surgery*.

